# Blockade of adrenergic β‐receptor activation through local delivery of propranolol from a 3D collagen/polyvinyl alcohol/hydroxyapatite scaffold promotes bone repair in vivo

**DOI:** 10.1111/cpr.12725

**Published:** 2019-11-20

**Authors:** Hao Wu, Yue Song, Junqin Li, Xing Lei, Shuaishuai Zhang, Yi Gao, Pengzhen Cheng, Bin Liu, Sheng Miao, Long Bi, Liu Yang, Guoxian Pei

**Affiliations:** ^1^ Department of Orthopaedics Xijing Hospital The Fourth Military Medical University Xi’an China; ^2^ Department of Orthopedic Surgery Linyi People’s Hospital Linyi China

**Keywords:** 3D printing, adrenergic β receptor, bone marrow stromal cells, bone regeneration, drug‐delivery system

## Abstract

**Objectives:**

Activation of the sympathetic system and adrenergic β‐receptors following traumatic bone defects negatively impairs bone regeneration. Whether preventing β‐receptor activation could potentially improve bone defect repair is unknown. In this study, we investigated the effect of systematic administration and local delivery of propranolol through composite scaffolds on bone healing.

**Materials and methods:**

Collagen/PVA/propranolol/hydroxyapatite（CPPH）composite scaffolds were fabricated with 3D printing technique and characterized by scanning electron microscope (SEM). Micro‐CT analysis and bone formation histology were performed to detect new bone formation. Osteogenic differentiation of bone marrow stromal cells (BMSCs) and osteoclastogenesis of bone marrow monocytes cultured with scaffolds extract were performed for further verification.

**Results:**

Intraperitoneal injection of propranolol did not significantly improve bone repair, as indicated by micro‐CT analysis and bone formation histology. However, CPPH scaffolds exhibited sustained release of propranolol in vitro and significantly enhanced bone regeneration compared with vehicle collagen/PVA/hydroxyapatite (CPH) scaffolds in vivo. Moreover, in vitro experiments indicated the scaffolds containing propranolol promoted the osteogenic differentiation and migration of rat BMSCs and inhibited osteoclastogenesis by preventing β‐receptor activation.

**Conclusions:**

This study demonstrates that local adrenergic β‐receptor blockade can effectively enhance the treatment of bone defects by stimulating osteogenic differentiation, inhibiting osteoclastogenesis and enhancing BMSCs migration.

## 1 INTRODUCTION

The incidence of high‐energy injuries continues to increase, and large bone defect cases are becoming more common in orthopaedic hospital.^1^ Bone defects exceeding a critical size do not possess self‐regeneration capacity and result in bone non‐union. Thus, large bone defects present a significant challenge to orthopaedic surgeons. Although autogenous bone grafting is considered, the “gold standard” approach for bone defect repair, donor site injuries and the limited availability of donor bone are serious disadvantages of this technique. 2 Therefore, the development of novel biomaterials and identification of potential therapeutic targets are urgently required to improve bone regeneration.

Major trauma and severe injury result in post‐trauma stress disorder, which activates the sympathetic system and induces a catecholamine surge.^3^, ^4^, ^5^, ^6^ In turn, this pathophysiologic reaction leads to host damage by activating adrenergic β‐receptors. 7, 8 Recently, a growing body of research has revealed that activation of β‐receptors negatively modulates bone remodelling under physiological conditions 9, 10 and impairs bone metabolism and bone fracture healing after trauma. 11, 12 Moreover, β‐receptors are expressed on osteoblastic cells and osteoclast‐like cells, and stimulation of β‐receptors using agonists inhibits alkaline phosphatase (ALP) activity and increases osteoclastic activity in vitro*.* 13, 14 This evidence indicates that the high levels of catecholamines induced by traumatic bone injury may activate adrenergic β‐receptors and negatively impact bone healing process. Therefore, adrenergic β‐receptors represent a potential therapeutic target to improve the treatment of bone defects.

Propranolol is a classic adrenergic beta‐blocker that exerts well‐characterized anti‐hypertensive effects. However, in‐depth studies have indicated that administration of propranolol enhances bone mineral density and reduces the risk of fracture in osteoporosis.^15^, ^16^ These positive clinical results promoted us to further explore the role of propranolol in the treatment of bone defects. However, limited studies that assessed the effects of systematic administration of propranolol on bone defect repair have reached conflicting conclusions.^17^, ^18^ Drug metabolism and clearance may make it difficult to achieve effective concentrations of the drug in the defect zone after systematic administration. Taking these limitations into consideration, local delivery of propranolol could potentially be more effective in the treatment of localized bone defects.

Local delivery system employs a carrier to deliver therapeutic molecules in order to improve the therapeutic potential by maintaining effective concentrations of the molecules or drug in the treatment area.^19^, ^20^ Our group previously constructed a porous bioactive collagen/hydroxyapatite scaffold that successfully improved bone regeneration.^21^ Polyvinyl alcohol (PVA) has been shown to release propranolol in a sustained manner in vitro and been employed as a release medium for the treatment of localized diseases such as infantile hemangioma.^22^, ^23^, ^24^ In this work, we added a low concentration of PVA incorporating propranolol to the raw collagen/hydroxyapatite homogenate to generate novel collagen/PVA/propranolol/ hydroxyapatite (CPPH) scaffolds. HPLC assays indicated the CPPH scaffolds continuously released propranolol in vitro for up to 3 weeks. Based on this stable drug‐release behaviour, we further investigated and evaluated the efficacy of local delivery of propranolol from CPPH scaffolds on bone regeneration in the rat critical‐sized femoral defect model. This study provides further knowledge of the effects and possible mechanisms of action of local β‐receptor blockade during the repair of critical‐sized bone defects in vivo.

## 2 MATERIALS AND METHODS

### 2.1 Fabrication of 3D collagen/PVA/hydroxyapatite scaffolds incorporating propranolol

Vehicle CPH scaffolds and CPPH scaffolds containing two ratios of propranolol were 3D printed using a filament‐free printing (FFP) printer (PC Printer, Particle Cloud Biotechnology, Xi'an, China). Hydroxyapatite powder (80‐100 nm‐diameter particles) was purchased from Emperor Nanomaterial Co. (Nanjing, China). Lyophilized type I collagen isolated from bovine Achilles tendon was purchased from Sannie Bioengineering Technology Co. (Tianjin, China). PVA was purchased from Sigma‐Aldrich (molecular weight, 85 000‐124 000 Da; degree of hydrolysis, 99%; St. Louis, MO, USA). Propranolol hydrochloride (99%, molecular weight, 295.8 Da) was purchased from Yuanye Bio‐Technology Co., Ltd.

In our previous work, a collagen‐hydroxyapatite (CHA) scaffold was printed using the inks (2 g of collagen, 4 g of HA, 10 mL of AAS) and successfully repaired bone defect of rabbits.^21^ We kept this ratio and added PVA to the inks for drug delivery. The printability was poor when the mass fraction of PVA solution was over 5 wt%, and the drug release was stable when PVA was 2wt% according to releasing patterns. Briefly, 2 g PVA was dissolved in 98 mL deionized water at 95°C to prepare 2 wt*%* PVA solution, and then, the solution was cooled to room temperature and various doses of propranolol (0, 10, 100 mg) were added to 1 mL of PVA solution to obtain 0%, 1% or 10% *w/v* propranolol solutions. Collagen (18% w/v) and 36% (w/v) hydroxyapatite were individually dissolved in 0.5 M acetic acid solution (AAS) and agitated to form turbid liquids. The printing ink was made of 1.8 g collagen, 3.6 g nano‐hydroxyapatite, 9 mL AAS and 1 mL PVA with propranolol. Air bubbles were removed from the CPH and CPPH (collagen/PVA/propranolol/hydroxyapatite) pastes using a vacuum, and the ink was transferred to the plotting cartridge and placed in the robotic deposition device.

The entire printing process was controlled by using the FFP software program installed in the printer. The STL profile encoding the scaffold characteristics was designed in advance and converted into G‐code. The ink was precisely delivered through the pinhole of the nozzle (diameter＝400 μm) and deposited onto the stainless‐steel baseplate at a constant *x*/*y*‐axes printing speed of 10 mm/s. To increase porosity and stability, each 200 μm thick layer was printed twice (back and forth; XXYY model) in the *z*‐axis to form a 400 μm story height.

Cubic scaffolds of different sizes were printed for the in vitro (10 mm × 10 mm × 3 mm) and in vivo (5 mm × 3 mm × 3 mm) tests. The CPH scaffolds without propranolol are called vehicle scaffolds in this study. The collagen/PVA/propranolol/hydroxyapatite ratio for the CPPH scaffolds was 1.8:0.02:0.01:3.6 w/w for the L‐Pro (low propranolol) scaffolds and 1.8:0.02:0.1:3.6 w/w for the H‐pro (high propranolol) scaffolds. The printed scaffolds were frozen at −80℃, lyophilized in a freeze dryer (Alpha 1‐2 LD plus, Christ, Osterode, Germany), immersed in 1% w/v genipin solution (Wako Pure Chemical Industries, Ltd., Kanagawa, Japan) for cross‐linking, sterilized using ethylene oxide and sealed in a sterile package until use.

### 2.2 Characterization of collagen/PVA/hydroxyapatite and collagen/PVA/propranolol/hydroxyapatite scaffolds

#### 2.2.1 Scanning electron microscopy and porosity analysis

The surface and microstructure of the 10 mm × 10 mm × 3 mm scaffolds were characterized by scanning electron microscopy (SEM, S‐4800; Hitachi, Tokyo, Japan). The porosity of the scaffolds was measured using a high‐resolution micro‐computed tomography (micro‐CT) analysis system (Yxlon) at 10.5 μm and 80 keV using VGStudio MAX software (Volume Graphics, Heidelberg, Germany).

#### 2.2.2 X‐ray diffraction analysis

The crystal phases of the scaffolds were scanned by X‐ray diffractometry (X’ Pert MPD PRO, PANalytical BV Netherlands) at a range of 10°‐80° at 2°/min and 3 kW.

#### 2.2.3 Mechanical properties of the scaffolds

Scaffolds (10 mm × 10 mm × 3 mm, n = 3) were soaked with phosphate‐buffered saline (PBS) for 24 h at 37℃ to simulate in vivo conditions. Stress relaxation testing was performed using a Model 858 Material Testing System (MTS System, Eden Prairie, MN, USA) at 0.3 mm/min until 10% of maximum compressive strain was reached.

#### 2.2.4 High‐performance liquid chromatography (HPLC)

Vehicle, L‐Pro and H‐Pro scaffolds were immersed in 5 mL PBS at 37°C and subjected to sustained oscillation using a vibrating plate. At the designated time points, aliquots of the solutions were collected and replaced with the same volume of PBS. All samples were stored at − 80℃ until the concentrations of propranolol in each solution were measured by high‐performance liquid chromatography (HPLC, Agilent Technologies, CA, USA). A standard propranolol solution (0.5 mg/mL) was used to determine the concentrations.

### 2.3 Biocompatibility of scaffolds

Cell viability was determined using the Cell Counting Kit‐8 assay (CCK‐8; Dojindo, Kumamoto, Japan) and annexin V‐FITC/propidium iodide (PI) staining (Liankebio Co., Hangzhou, China). The adhesion morphologies of BMSCs seeded on scaffolds were assessed via SEM. At specific time points, the scaffolds were fixed in 3% *v/v* glutaraldehyde at 4°C overnight, then dehydrated in a graded ethanol series, critical‐point dried, sputtered with gold and subjected to SEM analysis.

### 2.4 In vivo evaluation of bone regeneration

#### 2.4.1 Rat critical‐size femoral defect model

All animal procedures were approved by the Institutional Animal Care Committee of Air Force Medical University. Male Sprague Dawley (SD) rats (12 weeks old, weighing 280 ± 24 g) were acclimatized to a specific pathogen‐free (SPF) environment. Animals were anesthetized using 4 mg/kg xylazine hydrochloride and 30 mg/kg of 2% (*w/v*) pentobarbital (Rongbai Biological Technology Co., Ltd, Shanghai, China) for surgery. Standardized cylindrical femoral defects (approximately 3.5 mm in diameter; 5 mm long internally and externally) were created in the distal femur under saline irrigation; the defects lay above the medial femoral condyle. The defects were implanted as described below. Bone wax was used to seal the tunnels, and the wounds were sutured in layers. The rats received perioperative antibiotics (50 kU/kg penicillin) by intramuscular injection and post‐operative medical care.

For the systematic propranolol delivery experiments, six animals were paired according to weight and then randomly allocated into one of two groups (n = 3). The defects in both groups were implanted with vehicle (CPH) scaffolds. The propranolol injection group was subcutaneously injected with propranolol (0.5 mg kg^−1^ day^−1^) 5 days per week, and the vehicle group was injected with an equivalent volume of normal saline. After 12 weeks, all animals were sacrificed with an overdose of ketamine hydrochloride (Hengrui Medicine Co. Ltd, Jiangsu, China) for subsequent examination.

For the local propranolol delivery experiments, 27 rats were divided into three groups (n = 9). The defects in the L‐Pro and H‐Pro groups were implanted with L‐Pro and H‐Pro CPPH scaffolds containing propranolol, respectively, and the defects in the vehicle group with CPH scaffolds. Three rats in each group were sacrificed 4, 8 and 12 weeks after surgery.

To achieve fluorescent double staining, rats were intramuscularly injected with tetracycline (80 mg/kg; Sigma‐Aldrich) 14 days before euthanasia and calcein (8 mg/kg; Sigma‐Aldrich) 3 days before euthanasia (at 12 weeks after surgery).

#### 2.4.2 Micro‐CT bone analysis

The femur samples (n＝6 per group) were dissected, fixed in 80% ethanol and subjected to micro‐CT scanning (Y. Cheetah; Yxlon, Hamburg, Germany). The region of interest (ROI) was a 3.5 × 3.5 × 5 mm^3^ cylindrical region in the middle of the tunnel defects along the same major axis. A total of 450 micro‐CT slices were acquired for each sample, and the projections were reconstructed using VGStudio MAX software (Volume Graphics, Heidelberg, Germany).

#### 2.4.3 Histological staining and immunohistochemistry

Serial 5 μm thick cross‐sections of the decalcified samples were subjected to Masson's trichrome staining, and serial sections were stained using a Tartrate‐Resistant Acid Phosphatase Kit (TRAP) Staining kit (Sigma‐Aldrich) to detect osteoclasts. TRAP^+^ cells appear dark red, and TRAP‐positive multinucleated cells containing three or more nuclei were counted as mature osteoclasts.

Ten μm thick sections were used for immunohistochemical staining, and paraffin sections were deparaffinized, subjected to antigen recovery, incubated with primary antibodies against tyrosine hydroxylase (dilution 1:500, GB11181; Servicebio, Wuhan, China) and leptin receptor (dilution 1:500, ab5593; Abcam, Cambridge, MA, USA), followed by horseradish peroxidase‐coupled secondary antibodies (Aspen, Guangzhou, China), and counterstained with haematoxylin. 200 μm thick sections were observed using a fluorescence microscope to calculate the mineral apposition rate (MAR).

#### 2.4.4 Quantification of epinephrine and noradrenaline in plasma

Plasma samples were isolated by centrifuging whole blood at 4℃ (1400 *g* for 10 min）and stored at −80℃. Commercial ELISA kits (Cloud‐Clone Corp., Houston, TX, USA) were used to measure the levels of noradrenaline (NE) and epinephrine (Epi) in plasma following the manufacturer's instructions; the plates were read at 450 nm.

### 2.5 In vitro verification of biomechanism

#### 2.5.1 Evaluation of osteogenic differentiation

Based on the in vitro release profiles, vehicle, L‐Pro and H‐Pro scaffolds (10 mm × 10 mm × 3 mm) were immersed in 5 mL of standard osteogenic induction media (Cyagen Biosciences, Inc, Santa Clara, CA, USA) for 24 h to prepare conditioned osteogenic media.

BMSCs were cultured in scaffold‐conditioned osteogenic media with or without isoproterenol (1 μM), and the Alkaline Phosphatase Color Development Kit (Beyotime Institute of Biotechnology) was used to measure ALP activity on day 7. Alizarin red S (ARS, Sigma‐Aldrich Co., Taufkirchen, Germany) staining was performed on day 14 to detect mineral nodules. Samples were processed for Western blotting using standard techniques and incubated with anti‐osterix (ab209484, Abcam) and anti‐osteopontin (ab8448, Abcam).

#### 2.5.2 Cell migration assays

Cell migration assays were performed using 8 μm pore size transwell cell culture inserts (Corning, Tewksbury, MA, USA). BMSCs were serum‐starved for 12 h and seeded onto the upper chamber in media without FBS; scratch wound assays were employed to objectively evaluate BMSCs migration. BMSCs were cultured until 100% confluent in six‐well plates, and then, the cell layer was scarped using a 200 μL sterile pipette tip to create a linear wound and washed three times with PBS. The media was replaced with scaffold‐conditioned media containing isoproterenol. After 12 h, the scratch wound closure was measured under the microscope. Cell migration between two scratch edges was calculated using Image‐Pro Plus 6.0.

Immunofluorescent staining of F‐actin and vinculin was also used to indirectly distinguish migrating cells. Vinculin, F‐actin and nuclei were stained using an anti‐vinculin antibody (ab129002, Abcam), fluorescent rhodamine‐phalloidin (Cytoskeleton Inc, Denver, CO, USA, USA) and DAPI (Sigma‐Aldrich Co., Taufkirchen, Germany), respectively.

#### 2.5.3 Detection of osteoclastogenesis

Bone marrow monocytes (BMMs) isolated from rat femurs were induced to differentiate towards osteoclasts by incubation in α‐MEM media supplemented with 50 ng/mL receptor activator of nuclear factor‐kappa B ligand (RANKL) (PeproTech, London, UK) and 50 ng/mL macrophage colony‐stimulating factor（M‐CSF）in the presence or absence of isoproterenol (1 μM). Osteoclast differentiation was confirmed using the Tartrate‐Resistant Acid Phosphatase Kit (Sigma‐Aldrich) as described in section 2.9. Formation of F‐actin rings, a marker of osteoclasts, was detected using rhodamine‐phalloidin fluorescent staining (Cytoskeleton, Denver, CO, USA); nuclei were counterstained with DAPI（Sigma‐Aldrich Co., Taufkirchen, Germany. To detect intracellular reactive oxygen species (ROS), BMMs cultured in conditioned or control media with isoprenaline were measured with the fluorescent oxidation‐sensitive probe 2’,7’‐dichlorodihydrofluorescein diacetate (DCFH‐DA; Genmed, Shanghai, China). Expressions of osteoclast‐related factors tartrate‐resistant acid phosphatase (Trap) and cathepsin k (Ctsk) were also determined by Western blotting using standard techniques and incubated anti‐Trap (ab96372, Abcam), anti‐Ctsk (ab19027, Abcam) and anti‐β‐tublin (ab6046, Abcam).

### 2.6 Statistical analysis

Quantitative data are presented as the mean ± standard deviation (SD). All experiments were performed with at least n = 3 samples in each group. After confirming the data were normally distributed using the Kolmogorov‐Smirnov test, the differences between groups were analysed using two‐sided Student's tests or one‐way ANOVA analysis of variance, followed by Turkey's post hoc test. The statistical analysis was calculated by SPSS 16.0 software, and the levels of significance were set at *P < .05.

## 3 RESULTS

### 3.1 Systemic blockade of adrenergic β‐receptors does not obviously improve bone repair

Collagen/PVA/hydroxyapatite (CPH/vehicle) scaffolds were implanted into distal femur defects under sterile conditions (Figure S1A), and the rats were intraperitoneally injected with propranolol or normal saline every day.

**Figure 1:**
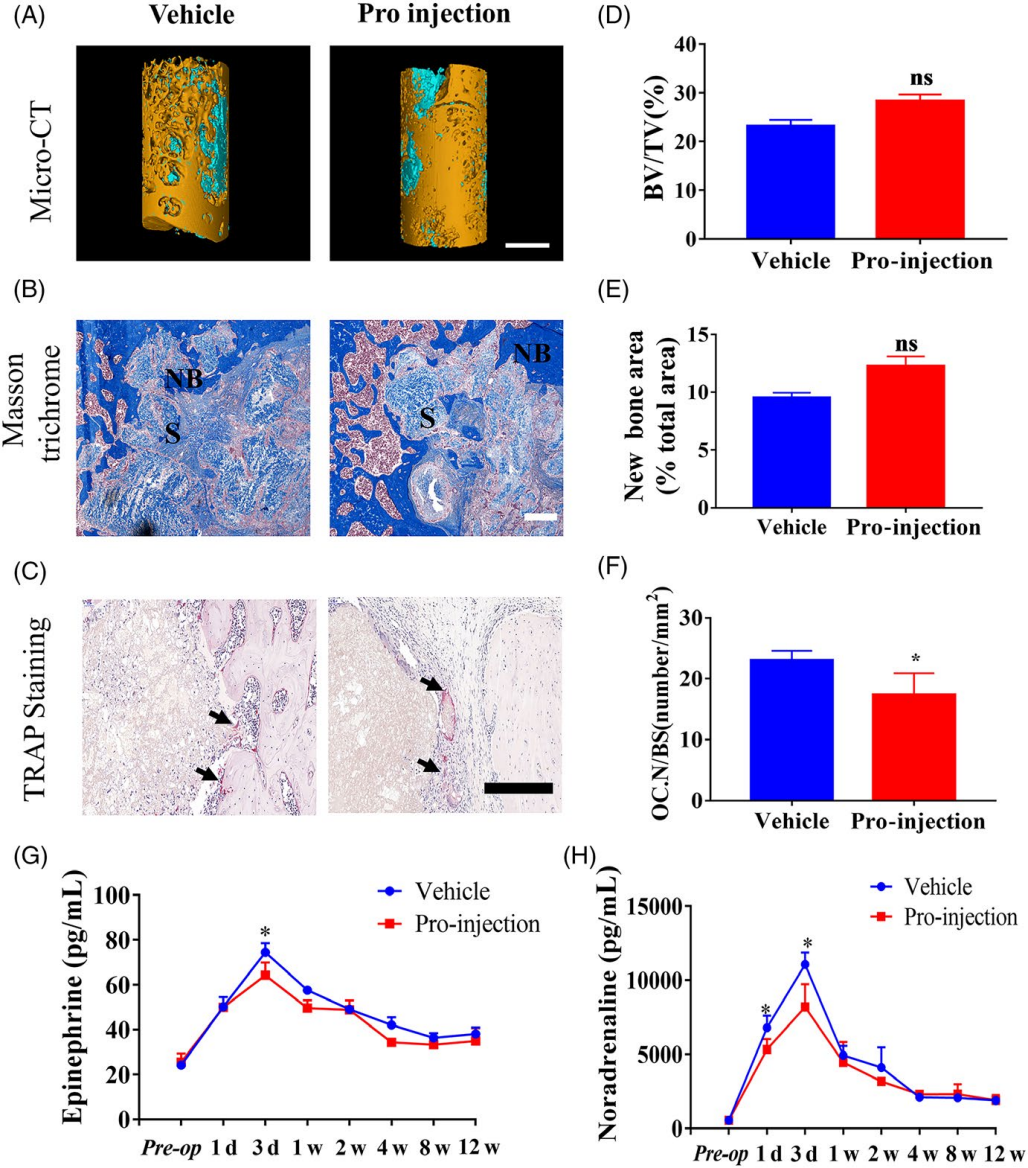
Evaluation of the effects of systemic administration of propranolol on bone repair in the rat critical‐size femoral defect model at 12 weeks. (A) Representative 3D micro‐CT reconstruction images of the defect zones in the femoral condyle. Scale bar: 2 mm. (B) Representative images of Masson trichrome staining in the defect zone. NB: new bone; S: scaffold; Scale bar: 200 μm. (C) Representative images of Trap staining. S: scaffold; B: bone; Black arrow: Trap + osteoclasts; scale bar: 200 μm. (D) Quantitative micro‐CT analysis of new bone volume (BV/TV) in the defect zone; n = 4 per group. (E) Semiquantitative analysis of the new bone area ratio in the defect zone; n = 4 per group. (F) Quantification of Trap + osteoclasts; n = 4 per group. (G‐H) Effect of surgery‐related trauma and propranolol injection on plasma epinephrine and noradrenaline levels; n = 3 at each time point. Data are mean ± SD; ns: not significant; *P < .05, **P < .01, ***P < .001. vs vehicle group injected with normal saline

Micro‐CT reconstructions of the defects at 12 weeks are presented in Figure 1A. New bone volume ratio (BV/TV) was slightly higher, but not significantly higher, in the propranolol‐treated group than the rats treated with normal saline (Figure 1D). Masson's trichrome staining and H&E staining confirmed that the area of decalcified new bone was not significantly greater in the group treated with propranolol (Figure 1B,E, Figure S1B,E). Injection of propranolol for 12 weeks slightly reduced the number of osteoclasts at the bone‐scaffold interface, as indicated by TRAP staining (Figure 1C,F), but did not significantly alter the mineral deposition rate (MAR) at 12 weeks (Figure S1C,F).

The plasma levels of catecholamine and noradrenaline were lower in the injection group within the first 3 days after surgery but not significantly different between groups from 1 week until 12 weeks after surgery (Figure 1G,H). TH^+^ sympathetic nerve density in the defect zone was not significantly different between groups at 12 weeks (Figure S1D,G).

### 3.2 Characterization of composite scaffolds containing propranolol

**Figure 2:**
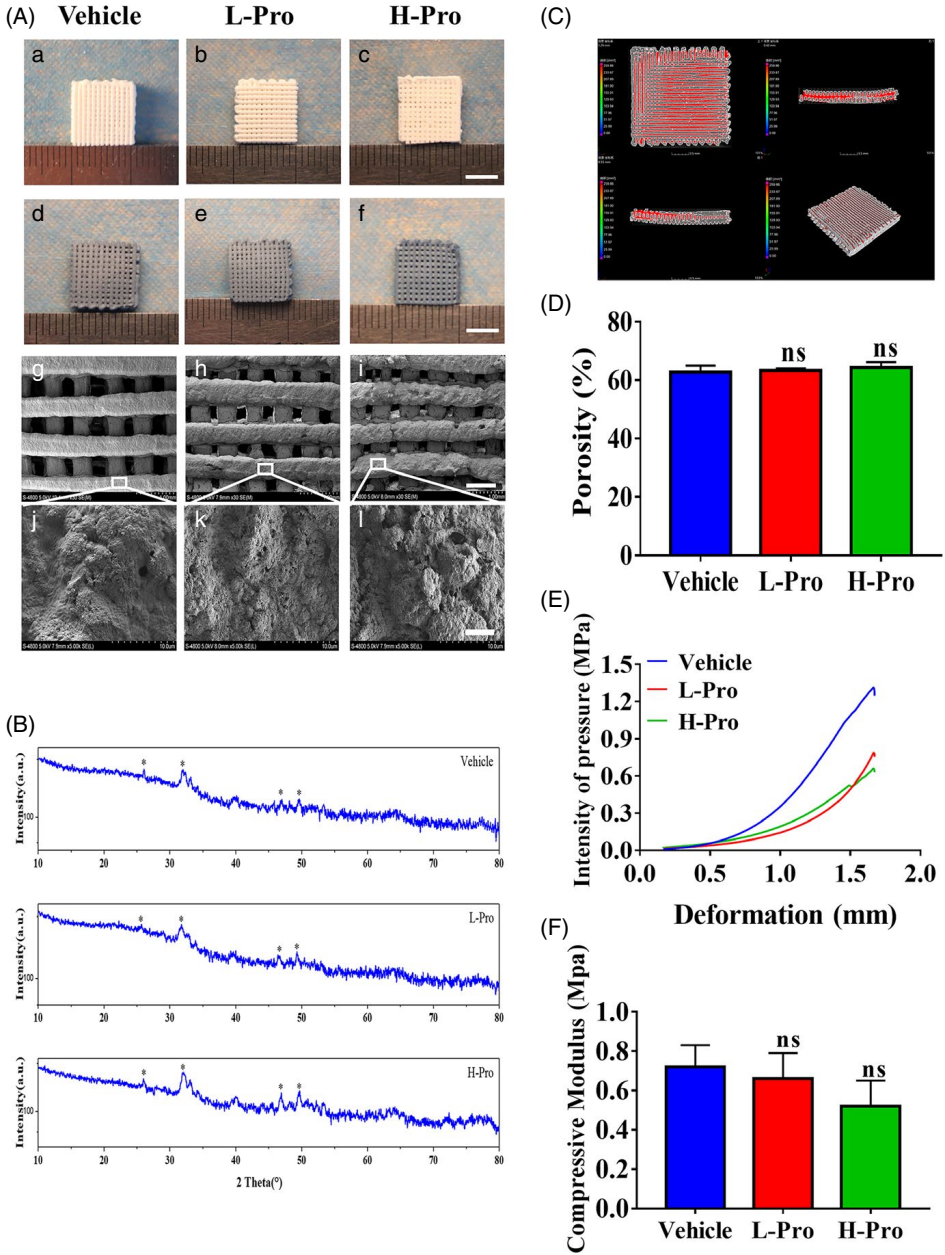
Characterization of propranolol‐loaded scaffolds. (A) Scaffolds incorporating different concentrations of propranolol (no propranolol/vehicle; low propranolol/L‐Pro; high propranolol/H‐Pro) were prepared by 3D printing, scale bar: 5 mm (a‐c); morphologic changes after cross‐linking the scaffold in genipin, scale bar: 5 mm (d‐f); scanning electron microscopy (SEM) images of cross‐linked scaffolds; magnification, 30×; scale bar: 0.2 mm (g‐i); microstructure of cross‐linked scaffolds observed by SEM; magnification, 5000×; scale bar: 5 μm (j‐l). (B) XRD patterns of vehicle/CPH and L‐Pro and H‐Pro scaffolds containing propranolol. Asterisk: peak of nano‐hydroxyapatite. (C) Micro‐CT reconstructions of cross‐linked scaffolds; (D) micro‐CT analysis of the porosity of cross‐linked scaffolds (n = 3). (E) Normalized compression curves for cross‐linked scaffolds. (F) Mechanical tests of the compressive moduli of cross‐linked scaffolds (n = 3). Data are mean ± SD; ns: not significant, *P < .05, **P < .01, ***P < .001. vs vehicle scaffolds

Uniform (10 × 10 × 3 mm^3^) vehicle, L‐Pro and H‐Pro scaffolds were fabricated via a 3D printing procedure. The scaffolds turned green after cross‐linking in genipin solution. SEM revealed that the surface of the scaffolds became rougher as the proportion of propranolol incorporated increased (Figure 2A). The average porosity of the cross‐linked scaffolds was determined by micro‐CT; the porosity values of the vehicle (62.71 ± 1.61%), L‐Pro (63.21 ± 0.57%) and H‐Pro (64.27 ± 1.35%) scaffolds were similar (Figure 2C,D). The peaks corresponding to nano‐hydroxyapatite were the major features of the XRD spectra of the vehicle, L‐Pro and H‐Pro scaffolds (Figure 2B). Compressive testing indicated that the addition of propranolol slightly reduced the mechanical strength of the scaffolds (Figure 2E,F), though the compressive moduli of the vehicle, L‐Pro and H‐Pro scaffolds were not significantly different (0.72 ± 0.09, 0.66 ± 0.11, 0.52 ± 0.11 MPa respectively; *P *> .05, n = 3).

### 3.3 Release patterns of propranolol in vitro

**Figure 3:**
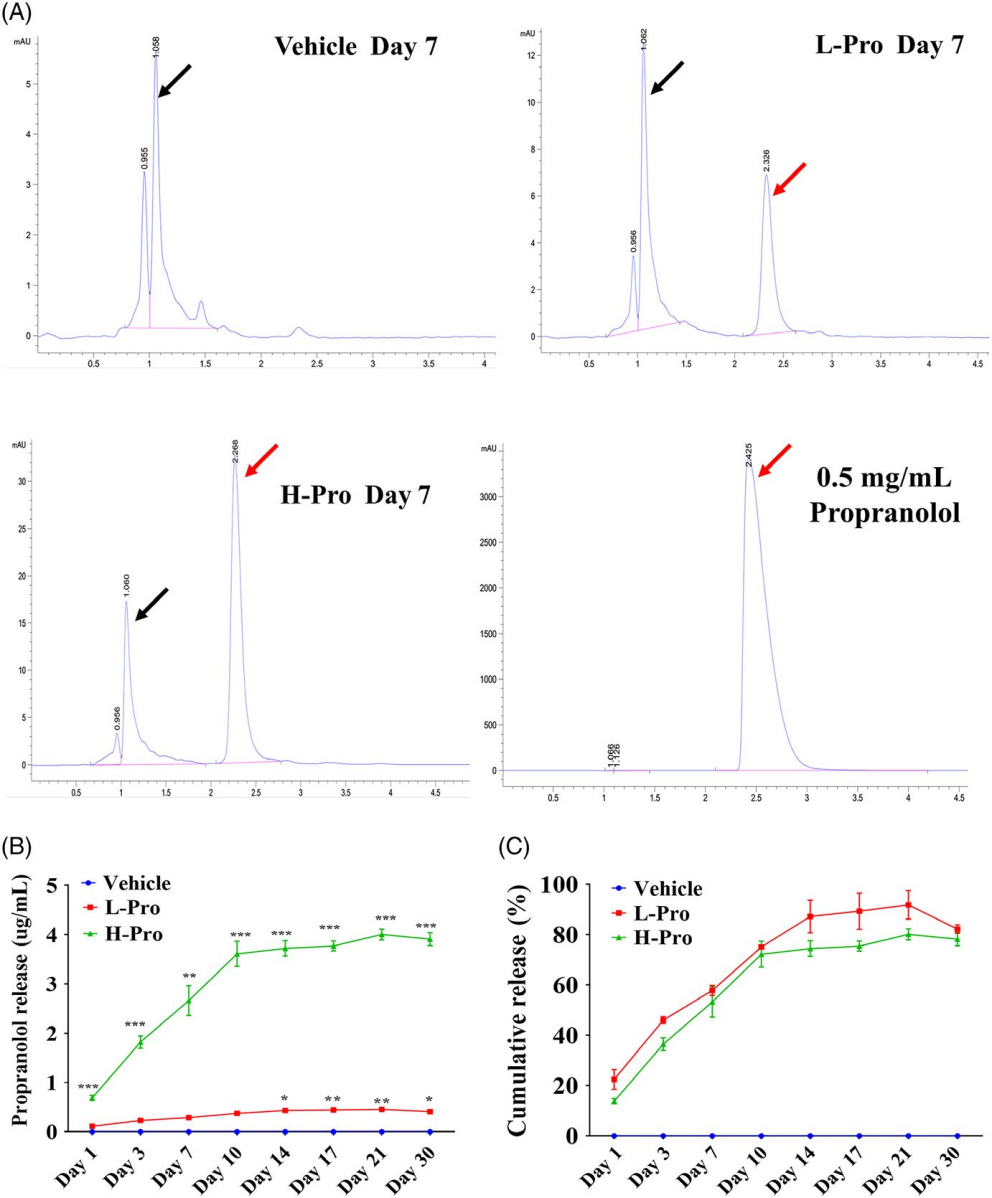
Release patterns of propranolol in the composite scaffolds. (A) High‐performance liquid chromatography analysis of propranolol released from scaffolds incorporating different concentrations of propranolol (no propranolol/vehicle; low propranolol/L‐Pro; high propranolol/H‐Pro) over 7 days immersed in PBS. Red arrow: propranolol in PBS, black arrow: scaffold components in PBS. (B and C) In vitro quantification of propranolol release kinetics for L‐Pro and H‐Pro scaffolds (n = 3 per time point). Data are mean ± SD; ns: not significant, *P < .05, **P < .01, ***P < .001. vs vehicle scaffolds

The scaffolds were immersed in 5 mL PBS for release assays, and the solutions were assessed by HPLC. The characteristic propranolol peak detected in the propranolol standard solution (0.5 mg/mL) was observed in the L‐Pro and H‐Pro solutions, confirming that propranolol was incorporated into and released by these scaffolds (Figure 3A). At 7 days, the concentration of propranolol released by the L‐Pro and H‐Pro scaffolds was 0.29 μg/mL and 2.67 μg/mL, respectively. The release curves indicated that propranolol was rapidly released from the L‐Pro and H‐Pro scaffolds over the first 14 days and then propranolol release gradually decreased. By day 21, approximately 91.8% and 80.1% of incorporated propranolol had been released from the L‐Pro and H‐Pro scaffolds, respectively (Figure 3B,C).

### 3.4 Adhesion and proliferation of BMSCs cultured with scaffolds in vitro

**Figure 4:**
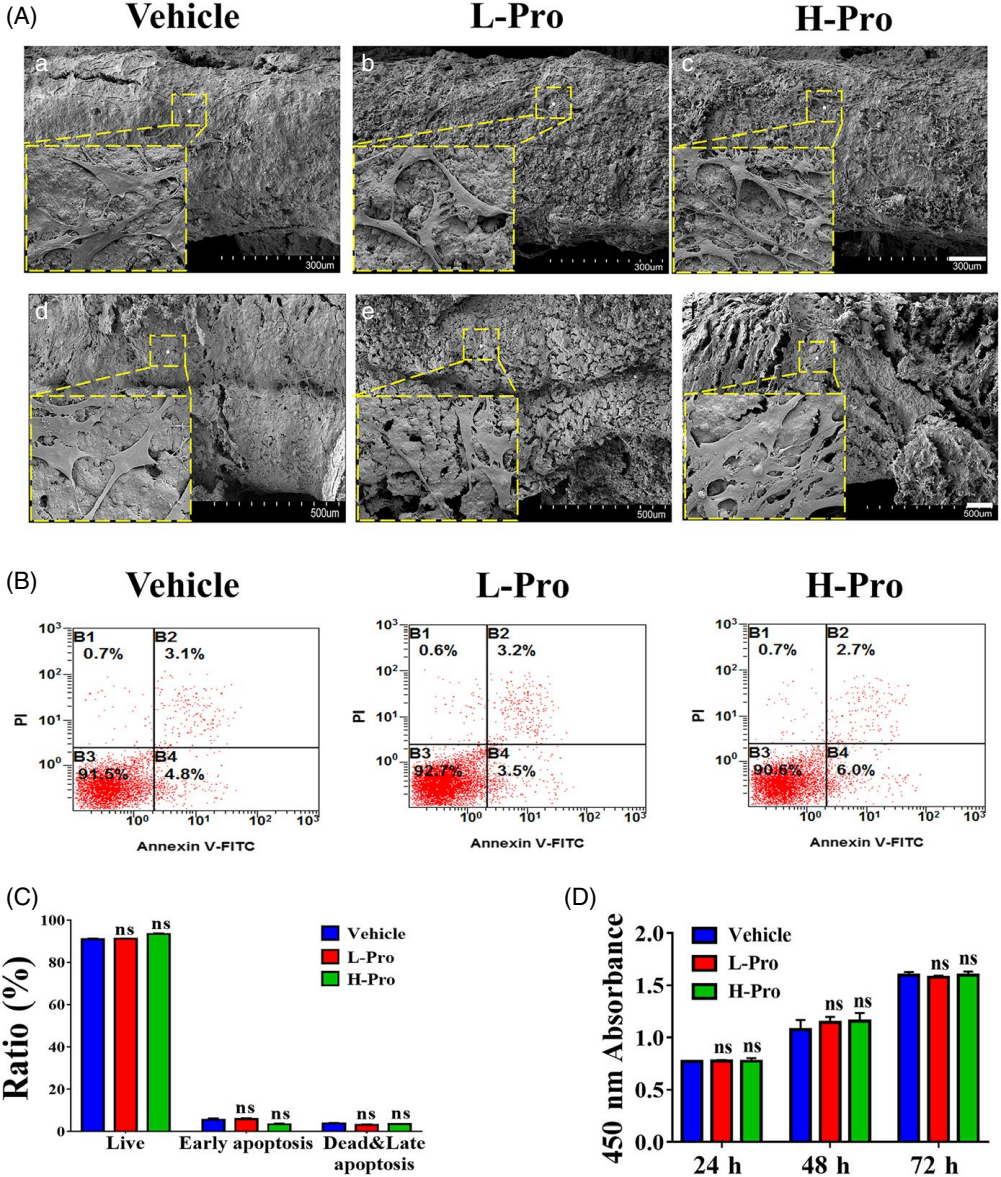
Assessment of the cytocompatibility of scaffolds containing propranolol towards BMSCs. (A) Representative SEM images of BMSCs adhesion on the beams of no propranolol/vehicle, low propranolol/L‐Pro and high propranolol/H‐Pro scaffolds at 7 days after seeding (a‐c); magnification, 150×; cross‐section of scaffolds showing growth of BMSCs into the pores (d‐f); magnification 100×; indicated zones are high‐power (500×) images. (B) Representative flow cytometry images and (C) quantification of the proportions of live and apoptotic BMSCs after culture in scaffold‐conditioned media for 24 h (n = 3). (D) CCK‐8 assay of the toxicity of scaffold‐conditioned media towards BMSCs. Data are mean ± SEM; ns: not significant; *P <0 .05, **P < .01, ***P < .001. vs vehicle scaffolds/vehicle scaffold‐conditioned media

BMSCs were seeded onto the surface of the scaffolds to examine the biocompatibility of the propranolol‐loaded scaffolds. After co‐culture for 24 h, the cells had begun to spread and occasionally adhere to the beams of the scaffolds (Figure S2). By 7 days, BMSCs covered most of the surface of the scaffolds (Figure 4A,A‐C). High‐power images (500×) indicated that cellular morphology varied between scaffolds. The cells on the vehicle scaffolds were flattened out and spread, whereas the cells on the L‐Pro and H‐Pro scaffolds exhibited long extended shapes with some rod‐shaped and granular structures (Figure 4A, S2). Scaffold profiles confirmed that numerous BMSCs invaded inside the interconnected pores of the scaffolds (Figure 4A,D‐F).

BMSCs were also cultured in scaffold‐conditioned media. Flow cytometry analysis revealed no significant differences in the ratios of living and apoptotic cells between groups (Figure 4B,C). The CCK‐8 assay confirmed that the BMSCs cultured in scaffold‐conditioned media proliferated over time, with no significant differences between groups at any time point (Figure 4D).

### 3.5 Propranolol released from scaffolds promotes bone formation and inhibits osteoclastogenesis in vivo

**Figure 5:**
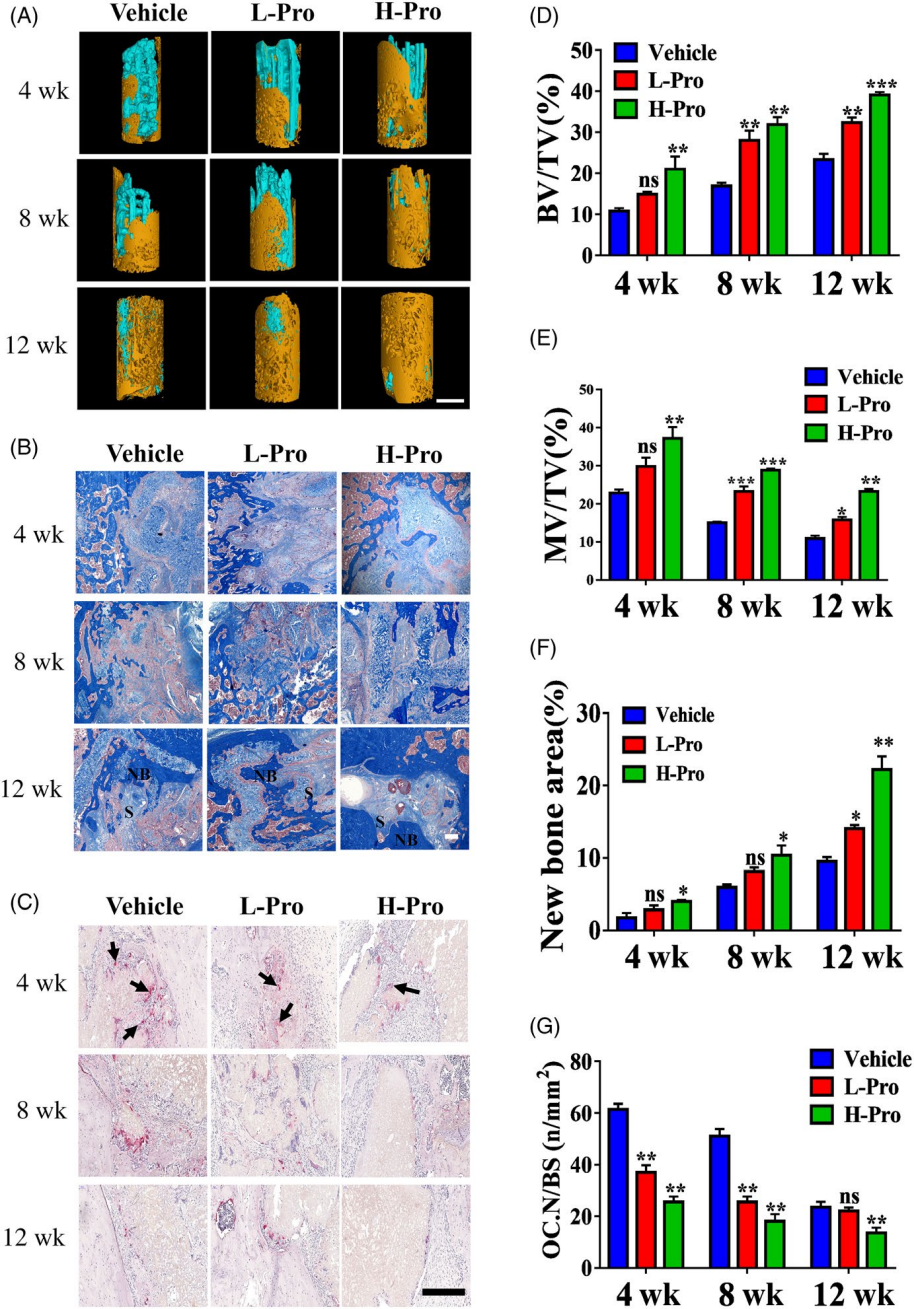
Evaluation of the effects of scaffolds containing propranolol on bone regeneration and osteoclastogenesis in the rat distal femoral defect model. The defects were implanted with no propranolol/vehicle scaffolds, low propranolol/L‐Pro scaffolds or high propranolol/H‐Pro scaffolds. (A) Micro‐CT reconstruction of bone formation in the defect zone at 4, 8 and 12 weeks. The yellow areas represent bone, and the blue/cyan areas indicate residual scaffold; scale bar: 2 mm. (B) Representative images of Masson's trichrome staining of new bone formation in the defect zone at 4, 8 and 12 weeks showing new bone (NB) and scaffold (S). Scale bar: 200 μm. (C) Representative images of Trap staining for osteoclasts at the interface between the scaffolds and new bone, the black arrows indicate the Trap + osteoclasts. Scale bar: 200 μm. (D‐E) Quantitative micro‐CT analysis of bone formation (BV/TV) and residue material (n = 3). (F) Semiquantitative analysis of new bone formation ratio in (B), (n = 3). (G) Quantification of Trap + osteoclasts (OC. N/BS), (n = 3). Data are mean ± SD. ns: not significant, *P < .05, **P < .01, ***P < .001. vs vehicle group

Due to the modest effects of systemic administration of propranolol on bone repair, we evaluated the impact of propranolol‐loaded scaffolds on bone repair in a 12‐week in vivo experiment. Rat critical‐sized femoral defects were implanted with vehicle, L‐Pro or H‐Pro scaffolds. 3D reconstructions of the scaffolds (blue) and bone tissue (yellow) and longitudinal sections are displayed in Figure 5A and Figure S3A. Quantitative micro‐CT analysis indicated significantly more bone formation (BV/TV) occurred in the groups implanted with the L‐Pro and H‐Pro scaffolds that incorporated propranolol compared with the vehicle scaffolds (Figure 5D).

Consistent with the micro‐CT results, histological analysis of new bone tissue using Masson's trichrome staining (blue) indicated that fresh bone tissue crossed the junction between the scaffolds and host bone at 4 weeks. Moreover, the histology demonstrated that large amounts of dense bone had formed by 12 weeks in the H‐Pro group, whereas only small amounts of new bony tissue were present in the vehicle group (Figure 5B,F). Additionally, the MAR was significantly higher in the L‐Pro and H‐Pro groups than the vehicle group (Figure S3B,C).

The residual scaffold volume was significantly higher in the L‐Pro and H‐Pro groups than the vehicle group at all time points, which indicates propranolol may inhibit scaffold resorption (Figure 5E). Trap staining revealed fewer Trap^+^ cells at the bone‐scaffold interface in the L‐Pro and H‐Pro groups than the vehicle group (Figure 5C,G). High‐power magnification images (100×) clearly identified Trap^+^ osteoclasts containing more than three nuclei (Figure S3D).

### 3.6 Scaffolds containing propranolol recruit BMSCs and impair inner sympathetic innervation during bone repair in vivo

**Figure 6:**
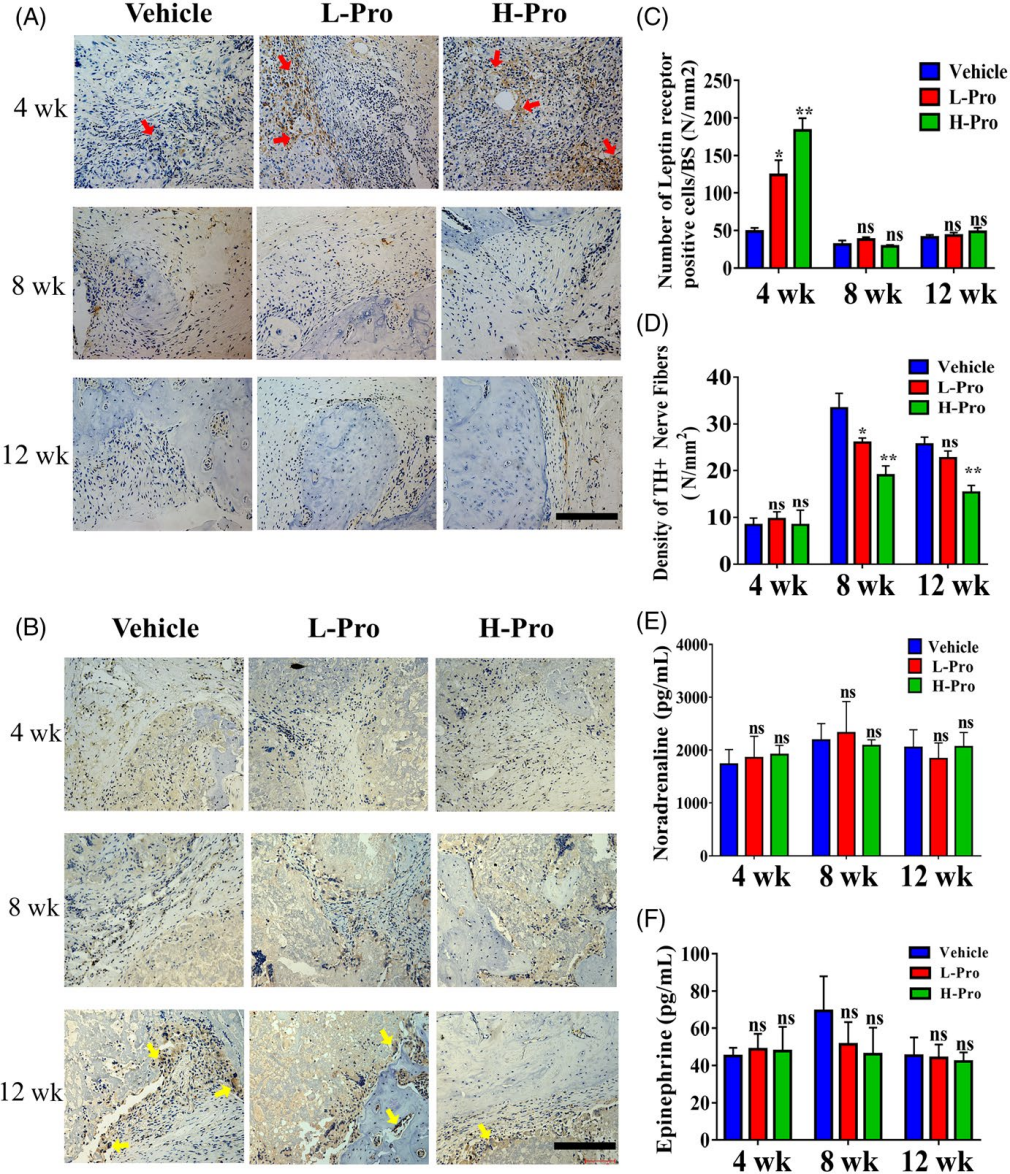
Evaluation of the effects of scaffolds containing propranolol on Leptin receptor+ （LepR+） BMSCs migration and the sympathetic activation in the rat distal femoral defect model. The defects were implanted with vehicle scaffolds, L‐Pro scaffolds or H‐Pro scaffolds. (A) Immunostaining for the Leptin receptor in the defect sites at 4, 8 and 12 weeks after scaffold implantation, red arrows indicate LepR + BMSCs. Scale bar: 200 μm. (B) Representative immunohistochemical images of TH + sympathetic nerves in the defect zone, yellow arrows indicate TH + nerves. Scale bar: 200 μm. (C) Semiquantitative analysis of LepR + cells in (A), n = 4. (D) Semiquantitative analysis of TH + nerve density in (B), n = 4. (E‐F) Baseline plasma epinephrine and noradrenaline levels in each group at 4, 8 and 12 weeks. Data are mean ± SD; ns: not significant, *P < .05, **P < .01, ***P < .001. vs vehicle group

Immunohistochemical staining for Leptin receptor (LepR^+^) demonstrated high numbers of LepR^+^ BMSCs were recruited into L‐Pro and H‐Pro scaffolds at 4 weeks. However, after this time point, the number of LepR^+^ stromal cells quickly diminished, with no significant difference between the L‐Pro, H‐Pro and vehicle groups at 8 and 12 weeks (Figure 6A,C, Figure S3E). However, these results indicate the scaffolds incorporating propranolol could recruit more BMSCs into the scaffolds, which may improve bone regeneration at the early stages of bone healing.

As shown in Figure 6B, tyrosine hydroxylase (TH^+^) adrenergic nerve fibres were rarely observed within the scaffolds at 4 weeks. As the scaffolds degraded and new bone in‐growth occurred, the total length of TH^+^ nerve fibres inside the scaffolds significantly increased (Figure 6B, Figure S3F). Semiquantitative analysis revealed the density of TH^+^ nerve fibres was lower in the H‐Pro (19.0 ± 1.63/mm^2^) and L‐Pro (25.7 ± 0.94/mm^2^) groups compared with the vehicle group (33.3 ± 2.62/mm^2^) at 8 weeks (Figure 6D).

The plasma levels of the catecholamines (epinephrine and noradrenaline) were not significantly different between the H‐Pro, L‐Pro and vehicle groups at 4, 8 and 12 weeks, indicating local delivery of propranolol had little impact on catecholamine circulation (Figure 6E,F).

### 3.7 Effect of propranolol scaffold‐conditioned media on osteogenic differentiation and migration of BMSCs in vitro

**Figure 7:**
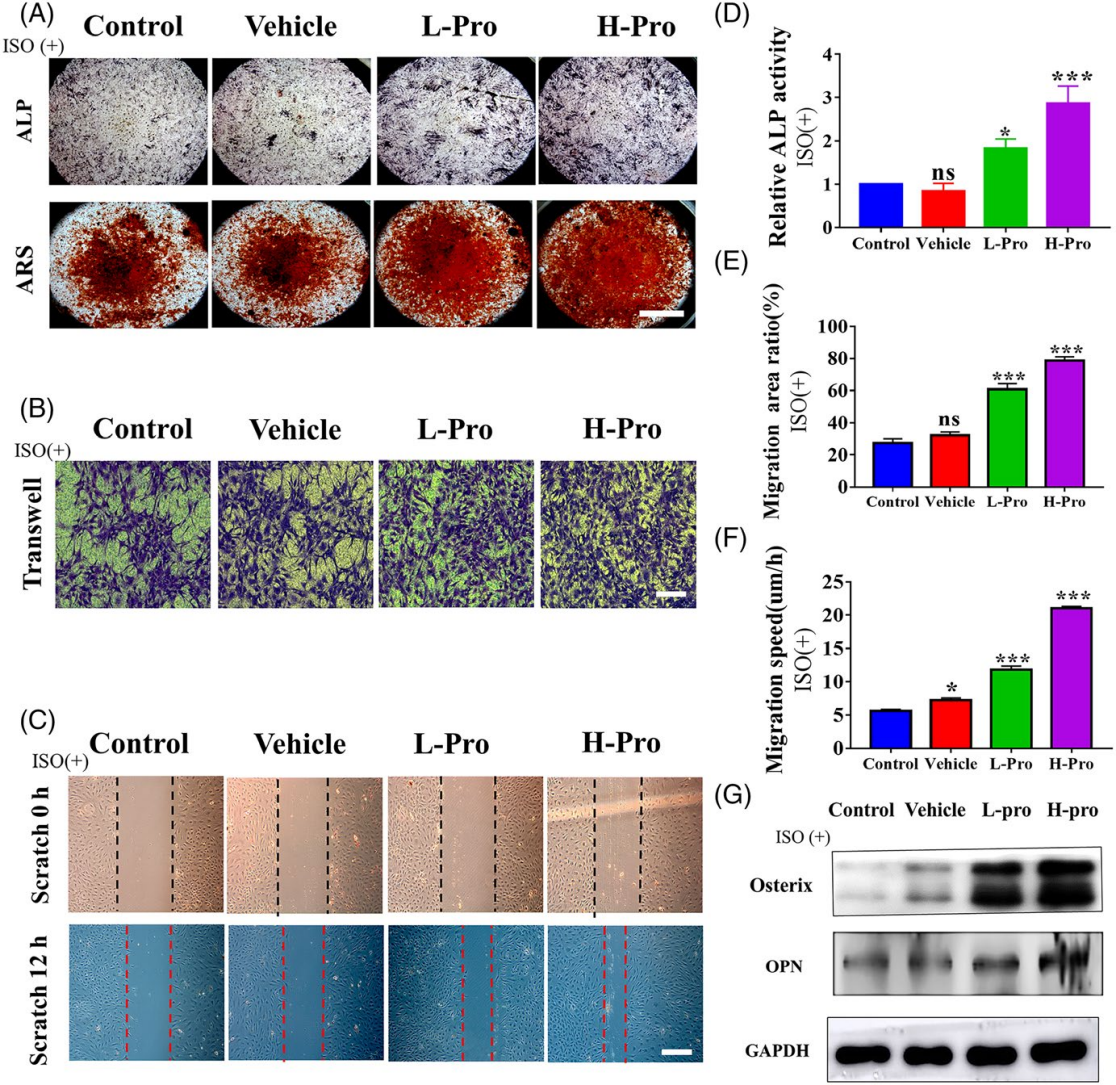
Effect of propranolol scaffold‐conditioned media on osteogenic differentiation and migration of BMSCs in vitro; ISO(+): β receptor agonist (isoprenaline). (A) ALP staining at 7 days and Alizarin Red S staining at 14 days, scale bar: 2 mm. (B) Effect of scaffold‐conditioned media on BMSCs migration in the transwell migration assay, scale bar: 200 μm. (C) Effect of scaffold‐conditioned media on BMSCs migration in the wound scratch assay; representative images of the dynamic wound healing process at 0 h and 12 h are shown, scale bar: 200 μm. (D) Relative ALP activity, n = 3. (E) Quantitative analysis of the migrated cell area ratio in the transwell migration assay (%), n = 3. (F) Calculation of the migration speed (μm/h) in the wound scratch assay, n = 3. Data are mean ± SD; ns: not significant, *P < .05, **P < .01, ***P < .001. vs control osteogenic media or control unconditioned media. (G) Western blotting of osteogenic markers osterix and osteopontin (Opn) expressions on day 7, n = 3

ALP staining and Alizarin Red S staining reminded us that osteogenic differentiation could not be influenced by propranolol in the absence of β activation, as well as cell migration shown in Transwell assay (Figure S4A‐D). After addition of the β‐adrenergic agonist isoprenaline, ALP activity and calcium deposition were clearly higher in the L‐Pro and H‐Pro groups (Figure 7A,D). *Osterix* and *Opn* expressions were also enhanced at 7 days in the presence of the β‐receptor agonist isoprenaline (Figure 7G). The area covered by migrated BMSCs was 27.4%, 32.2% (*P *> .05), 60.9% (*P *< .001) and 78.6% (*P < *.001) in the control, vehicle, L‐Pro and H‐Pro groups, respectively (Figure7B,E). The wound scratch assay showed that the rate of cell migration was higher in the L‐Pro (11.8 ± 0.46 μm/h, *P *< .001) and H‐Pro (21.1 ± 0.25 μm/h, *P *< .001) groups than the control (5.6 ± 0.17 μm/h) and vehicle groups (7.25 ± 0.29 μm/h Figure 7C,F). Immunofluorescent staining for F‐actin and vinculin demonstrated fewer focal adhesions—indicative of a migratory phenotype—were present in the BMSCs in the L‐Pro and H‐Pro groups than in the vehicle and control groups (Figure S4E,F).

### 3.8 Effect of propranolol scaffold‐conditioned media on osteoclasts formation in vitro

**Figure 8:**
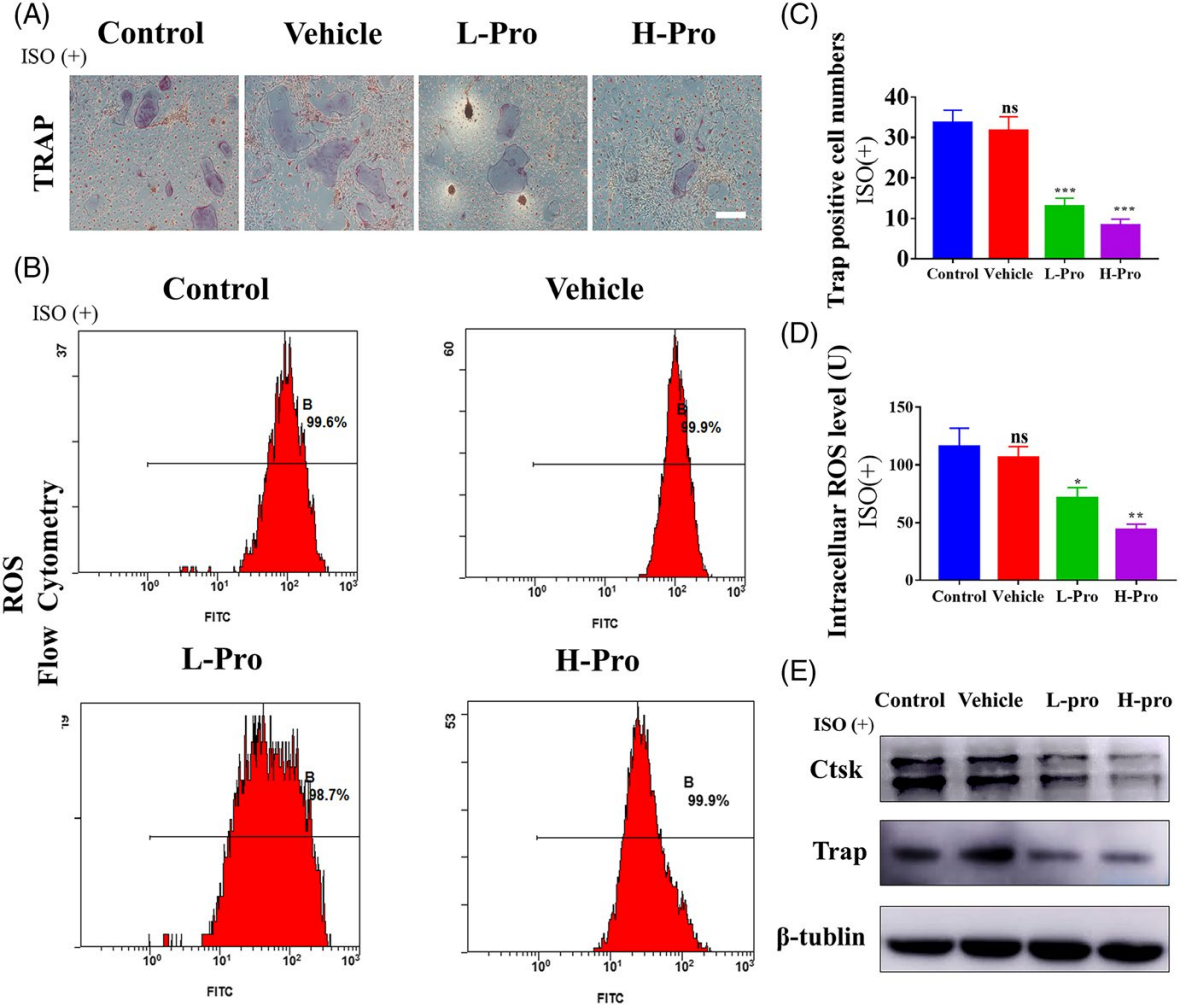
Effect of propranolol scaffold‐conditioned media on osteoclastogenesis in vitro. ISO(+): β receptor agonist (isoprenaline) positive. (A) Trap staining for osteoclasts cultured with isoprenaline, scale bar: 200 μm. (B) Flow cytometry analysis of intracellular reactive oxygen species using the oxidation‐sensitive fluorescent probe 2’,7’‐dichlorodihydrofluorescein diacetate (DCFH‐DA). (C) Quantification of Trap + osteoclasts with more than three nuclei, n = 3. (D) Quantification of intracellular ROS levels, n = 3. (E) Western blotting of osteoclast‐related gene Ctsk and Trap expressions on day 5. Data are mean ± SD; ns: not significant, *P < .05, **P < .01, ***P < .001. vs control unconditioned media

Trap staining indicated that propranolol inhibited osteoclast formation by preventing activation of β‐receptors (Figure 8A,C, Figure S5A,B). Western blotting also revealed when the β‐receptors were activated, and *Trap* and *Ctsk* expressions were lower at 5 days in the L‐Pro and H‐Pro groups compared with the control and vehicle groups (Figure 8E). In addition, the inhibition of osteoclastogenesis in L‐Pro and H‐Pro groups was associated with reduced ROS levels, rather than inhibition of BMMs proliferation (Figure 8B,D, Figure S5C,D).

## 4 DISCUSSION

Current evidence suggests activation of the sympathetic system impairs bone formation and remodelling via adrenergic β‐receptors, and blocking β‐receptor activation positively influences bone homeostasis.^25^, ^26^ As a common clinical beta‐receptor blocking agent, propranolol has been suggested as a potential treatment for osteoporosis and may also reduce the risk of fracture.^15^, ^27^ In our initial exploration, systematic injection of propranolol did not improve the volume of bone formation after implantation of collagen/PVA/hydroxyapatite scaffolds. Subaie et al (2016) and Minkowitz et al (1991) found that systematic administration of propranolol could enhance endochondral bone formation and osteointegration of implants. The conflict between our results and those studies may be due to the different modes of repair in those bone defect models, which can self‐repair without implantation of scaffolds.^17^, ^28^

**Scheme 1:**
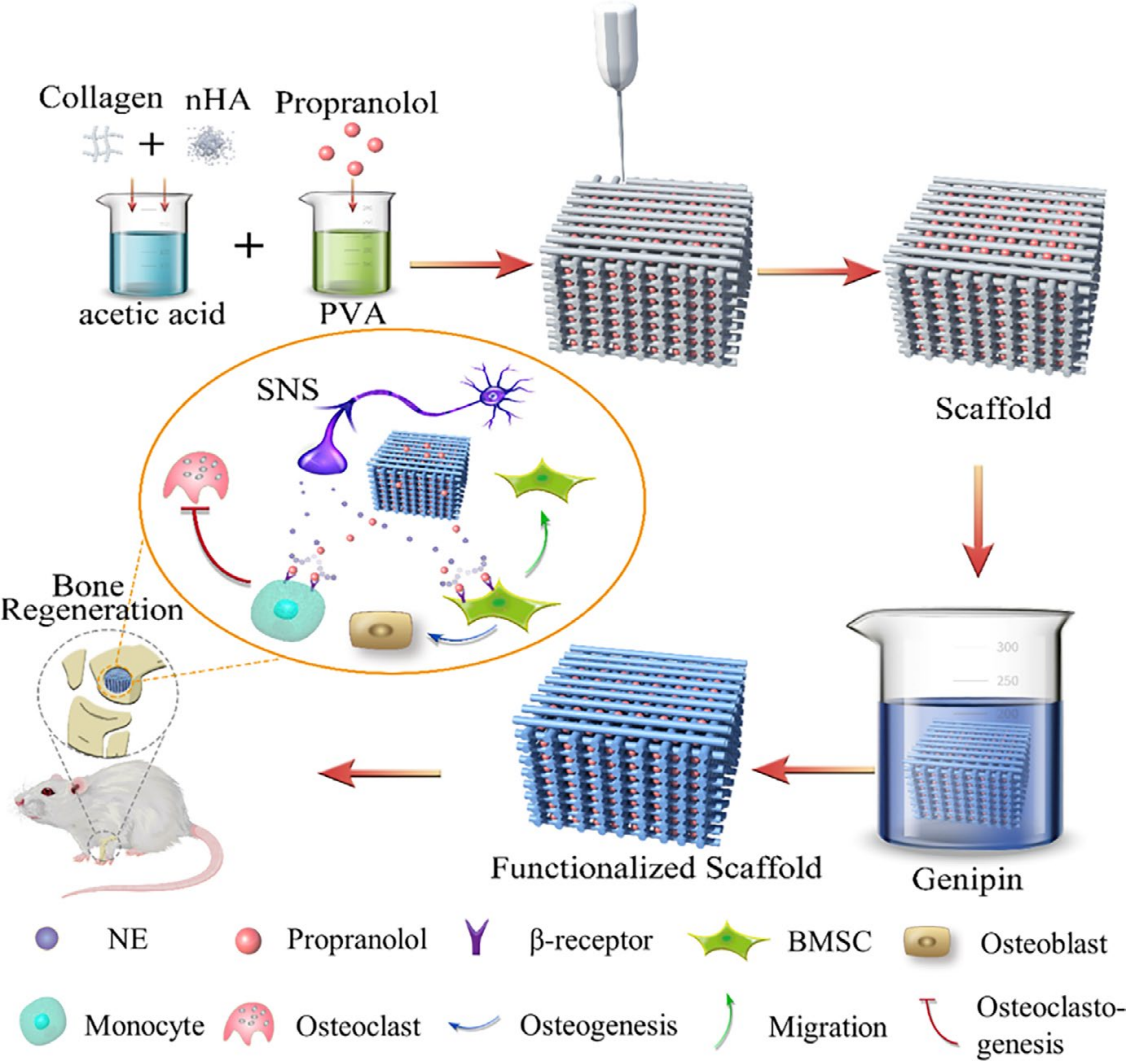
Schematic diagram of CPPH (collagen/PVA/propranolol/hydroxyapatite) biocomposite scaffold fabrication and treatment of bone defect

Delivery of a drug directly to the defect site using a local delivery system may improve the treatment effects in bone regeneration.^29^, ^30^ To prolong the release of propranolol, we constructed collagen/PVA/propranolol/hydroxyapatite scaffolds via a 3D printing technique. This novel scaffold exhibited good mechanical properties and biocompatibility with BMSCs. Encouragingly, after functionalization with the natural cross linker genipin, in vitro kinetic release assays showed the scaffolds led to sustained release of propranolol for at least 3 weeks.^31^, ^32^ The concentrations of propranolol released into 5 mL PBS after 24 h by the L‐Pro and H‐Pro scaffolds were approximately 0.12 and 2.18 μM, respectively, and a previous study reported that 1 μM propranolol reversed the inhibitory effects of isoprenaline on the osteogenic differentiation of BMSCs in vitro.^33^ These results indicate that the scaffolds release pharmacologically relevant concentrations of propranolol in vitro (Scheme 1).

Larger areas of new bone tissue formed inside the scaffolds containing propranolol compared with vehicle scaffolds. Trap staining in vivo demonstrated that locally released propranolol strongly inhibited osteoclastogenesis, which is critical for effective bone remodelling and repair.^34^ As shown in Figure 6A, L‐Pro and H‐Pro scaffolds contained higher numbers of leptin receptor^+^ cells than the vehicle scaffolds at 4 weeks, but not at 8 and 12 weeks post‐implantation. Recruitment of endogenous mesenchymal stromal cells to the trauma site is also required to initiate bone regeneration.^35^ Leptin receptor^+^ mesenchymal stromal cells, a major subpopulation of BMSCs derived from the periosteum, migrate to the fracture zone and proliferate rapidly in response to fracture or injury .^36^ Recent studies suggest the sympathetic nerves are a component of the bone marrow niche, while degeneration of adrenergic nerves increases the abundance of BMSCs in the bone marrow. Du et al (2014) demonstrated that denervation of sympathetic nerves also increased stromal cell migration.^37^, ^38^ We speculated that the propranolol‐releasing scaffolds increased the recruitment of BMSCs during the early stages of bone repair and that these recruited stromal cells subsequently proliferated and differentiated into osteoblasts to form new bone tissue. To our surprise, scaffolds loaded with propranolol were innervated with fewer sympathetic nerve fibres. These results suggest that locally blocking β‐receptors may modulate chemorepulsive guidance cue production such as semaphorin 3A (Sema3A) and thus prevent sympathetic innervation.^39^

The in vitro results indicated the ability of propranolol to affect osteoblast differentiation was related to antagonism towards isoprenaline. Majeska et al (1992) found that culturing osteoblasts with isoprenaline massively increased cyclic adenosine monophosphate (cAMP) levels and inhibited ALP activity, while propranolol counteracted these effects of isoprenaline by more than 60%.^13^ In terms of osteoclast formation, isoprenaline is well characterized to increase ROS generation, which directly promotes osteoclast formation.^40^, ^41^ Consistent with increased LepR^+^ BMSCs recruitment to L‐Pro and H‐Pro scaffolds in vivo, blocking β‐receptor activation using propranolol scaffold‐conditioned media also accelerated the migration of BMSCs in vitro. Based on our in vitro assays, we conclude that local blockade of adrenergic β‐receptors using propranolol leads to a combination of effects during bone reconstruction, including increased osteogenesis and BMSCs recruitment and reduced osteoclastogenesis.

However, there are some limitations to this study. The in vitro assays showed the propranolol scaffolds promoted bone regeneration by competing with isoprenaline stimulation. Therefore, the role of exogenous isoprenaline during bone repair needs to be studied in vivo. Moreover, the density of adrenergic β‐receptors and catecholamine levels in the defect zone should be quantified to clarify how propranolol alters the sympathetic system inside scaffolds. Finally, sympathetic nerve innervation varies in different types of bone and between species, so further studies on different bones and in larger animals are necessary to validate our findings.

In conclusion, this study demonstrates that—in contrast to systemic administration—local adrenergic β‐receptor blockade can effectively enhance the treatment of bone defects and excellent therapeutic effects can be achieved using 3D‐printed composite scaffolds. In the future, we believe that scaffolds incorporating factors that block sympathetic activation will hold significant potential for bone tissue engineering applications.

## CONFLICT OF INTEREST

The authors have no competing financial interests to declare.

## AUTHOR CONTRIBUTIONS

GP, LY, HW and LB conceived and designed the research; HW, YS, JL and SM carried out the experimental work; BL provided the GFP transgenic SD rats; HW, XL, SZ, YG and PC analysed the data; HW, YS wrote the paper; all authors read and approved the final manuscript. Hao Wu, Yue Song and Junqin Li should be considered joint first author.

## Data Availability

All data that support the findings of this study are available from the corresponding author upon reasonable request.
