# Supplementary material for: Blockade of adrenergic β‐receptor activation through local delivery of propranolol from a 3D collagen/polyvinyl alcohol/hydroxyapatite scaffold promotes bone repair in vivo
Source: Cell Prolif. 2019 Nov 20;53(1):e12725. doi: 10.1111/cpr.12725 (PMC6985692; doi:10.1111/cpr.12725)
Supplement: Supplementary file 1 [file CPR-53-e12725-s001.docx]

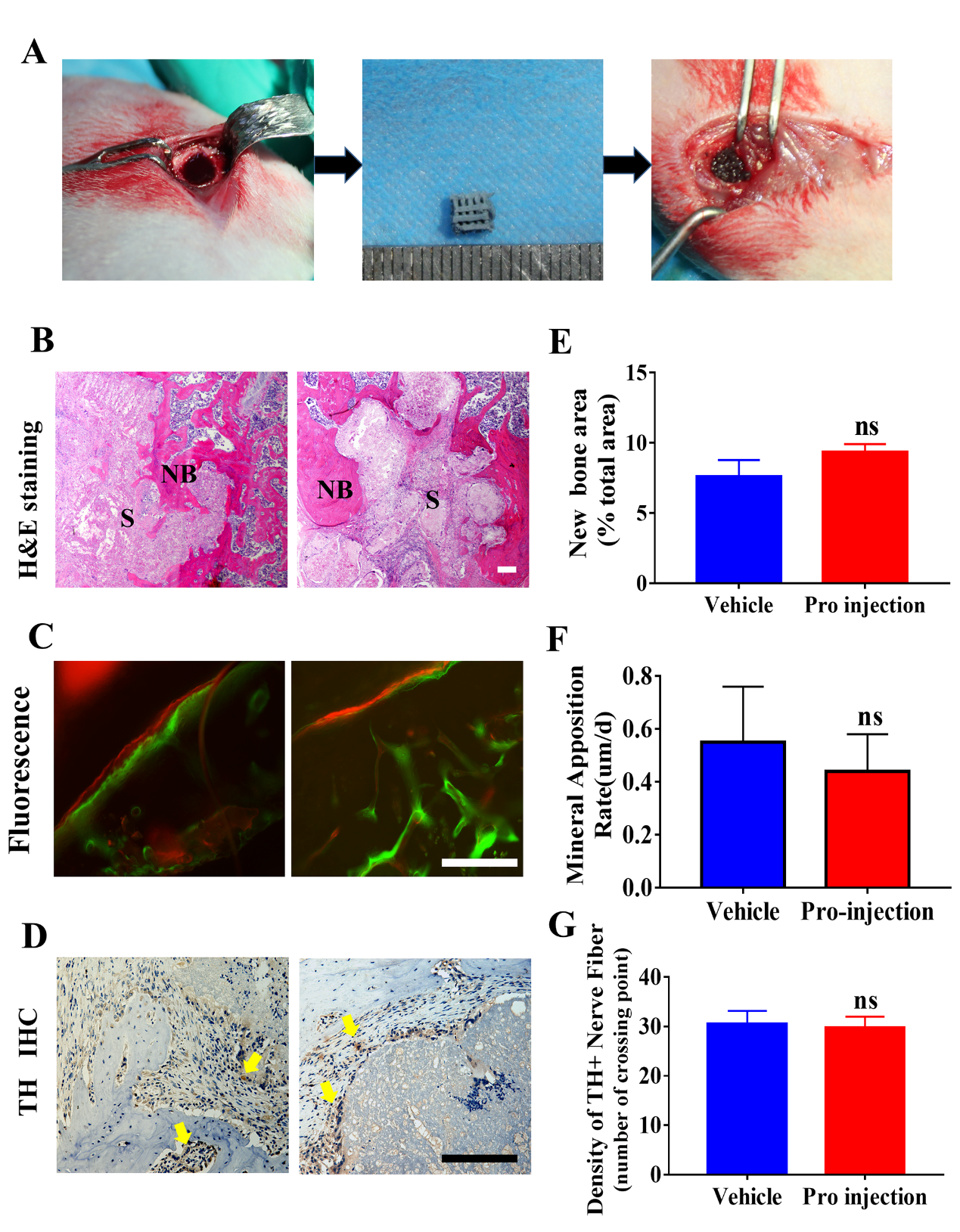


**Figure S1.** (A) Representative images of the surgical procedure. (B) H&E staining of bone regeneration in the defect zone at 12 weeks. (C) Fluorochrome double-labeling of Alizarin red (red) and calcein (green) in the defects at 12 weeks. (D) Immunohistochemical images of TH^+^ sympathetic nerves in the defect zone at 12 weeks. (E) Semiquantitative analysis of new bone area (%) at 12 weeks, *n* = 4. (F) Mineral apposition rate (MAR) of new bone formation, *n* = 4. (G) Semiquantitative analysis of TH^+^ nerve density in (C), *n* = 4. Scale bar: 200 μm. Data are mean ± SD; ns: not significant; **p* < 0.05, ***p* < 0.01, ****p* < 0.001. vs. vehicle scaffolds.


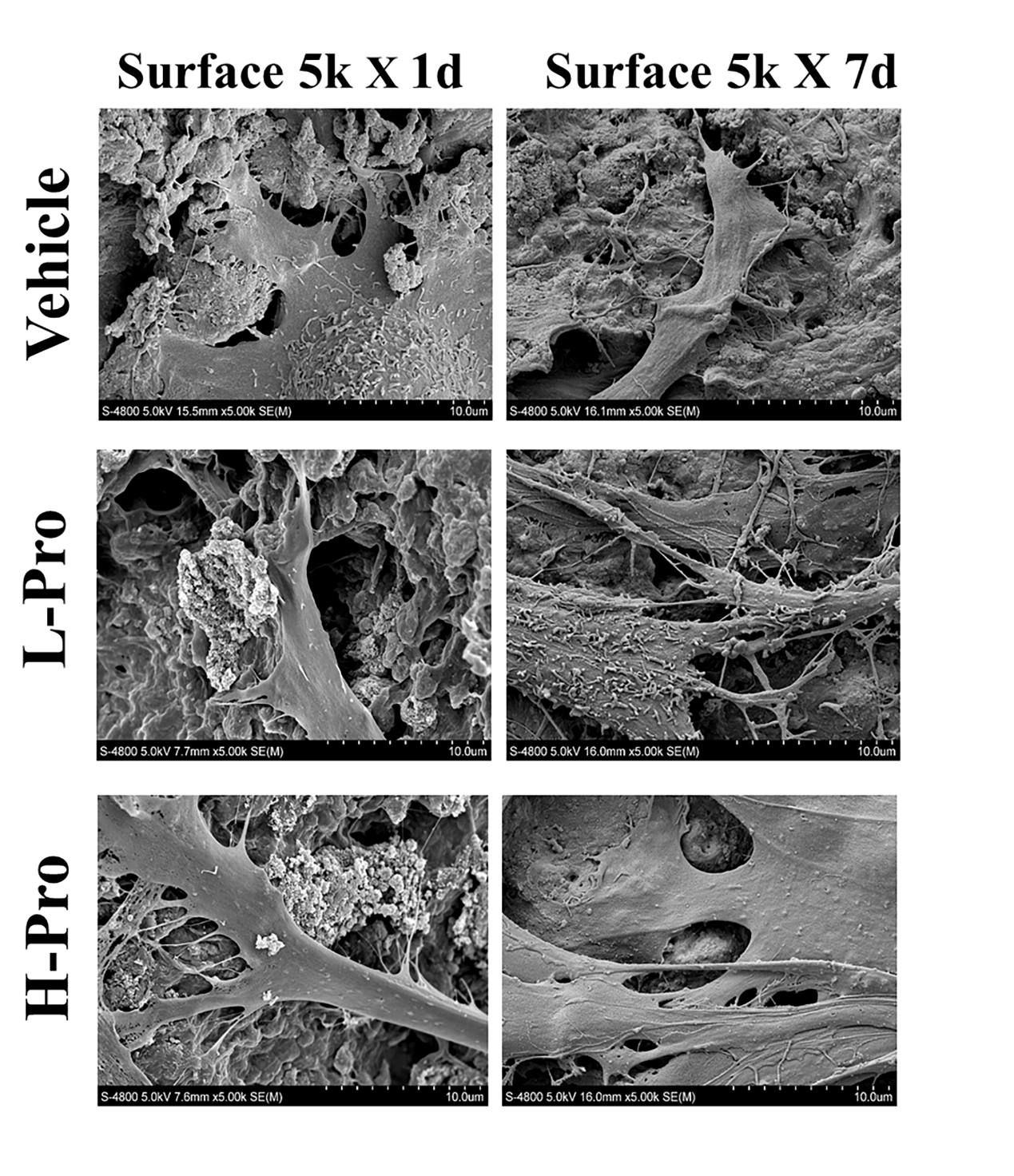


**Figure S2.** Representative SEM images of BMSC adherence to vehicle/CPH and L-Pro and H-Pro scaffolds containing propranolol at 1 d and 7 d after seeding.


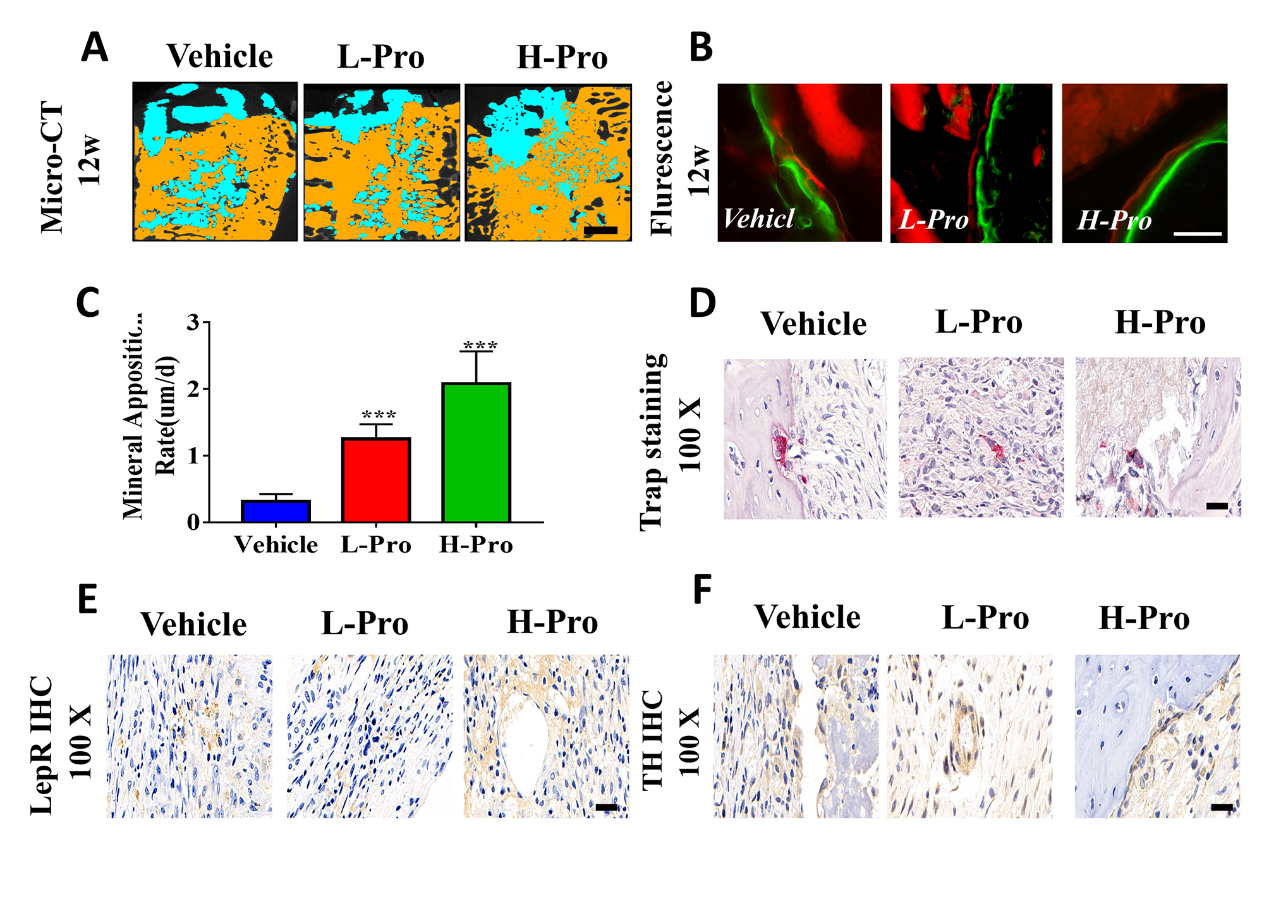


**Figure S3.** (A) Representative micro-CT reconstructions of vertical scaffold sections at 12 weeks. Scale bar: 2 mm. (B) Fluorochrome double-labeling of new bone formation in the defect zone. Scale bar: 200 μm. (C) Mineral apposition rate of new bone formation at 12 weeks, *n* = 4. (D) Observation of typical Trap^+^ osteoclasts in the defect zone; 100×; scale bar: 20 μm. (E) Immunostaining of Leptin receptor^+^ BMSCs at the magnification of 100×, scale bar: 20 μm. (F) Representative immunohistochemical images of TH^+^ cells; 100×; scale bar: 20 μm. Data are mean ± SD. ns: not significant; **p* < 0.05, ***p* < 0.01, ****p* < 0.001. vs. vehicle scaffolds.

**
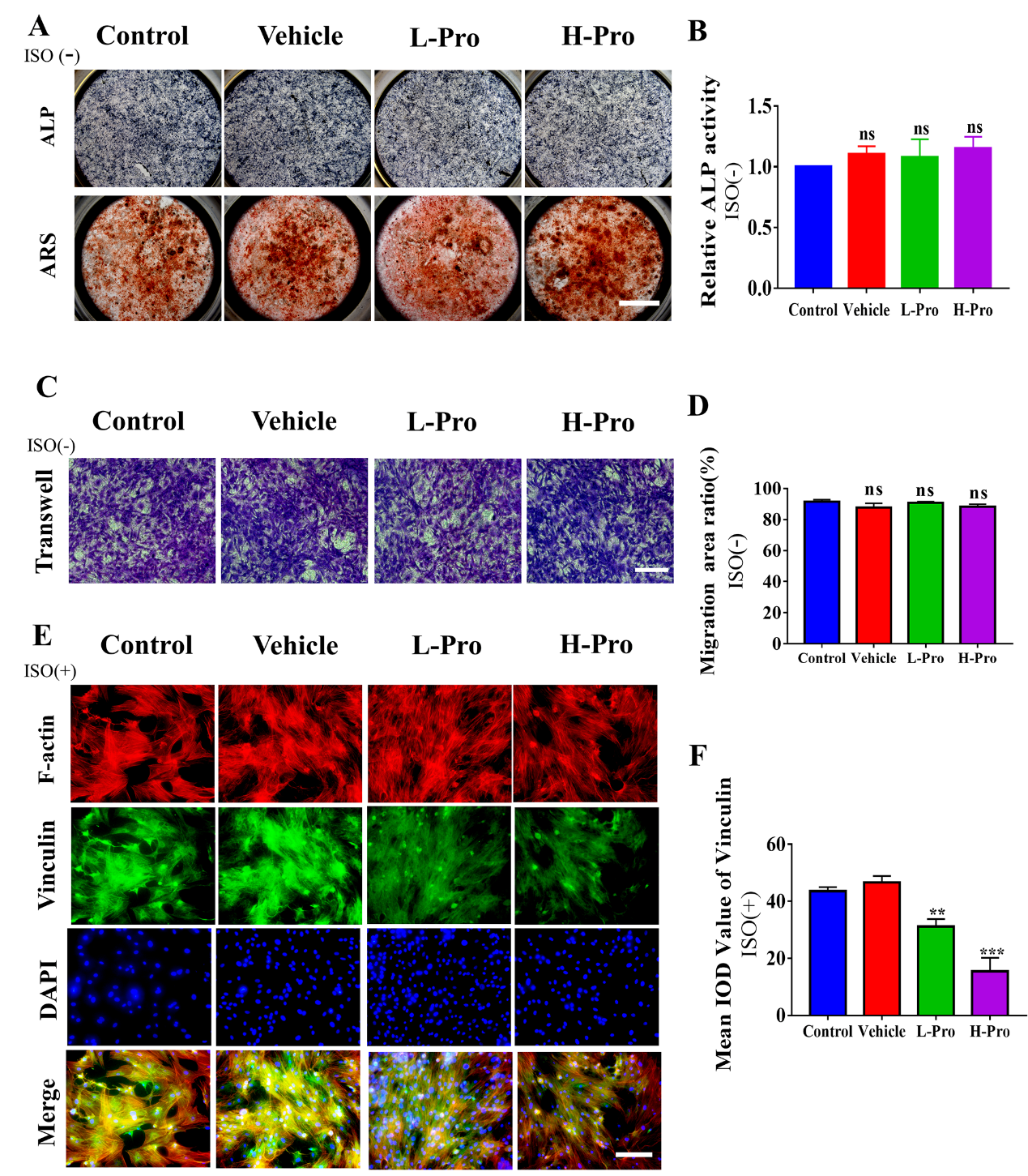
**

**Figure S4.** (A) ALP staining at 7 days and Alizarin Red-S staining at 14 days of BMSCs cultured in scaffold-conditioned media without isoprenaline. Scale bar: 2 mm. (B) Measurement of relative ALP activity, *n* = 3. (C) Transwell migration assay of BMSCs in scaffold-conditioned medium without isoprenaline. Scale bar: 200 μm. (D) Quantitative assessment of migrated cell area ratio (%) in (A), *n* = 3. (E) Representative images of BMSCs immunostained with F-actin and vinculin after culture in scaffold-conditioned media for 24 h, scale bar = 100 μm. (F) Integral optical density values of vinculin expressed by BMSCs. Data are mean ± SD. ns: not significant; **p* < 0.05, ***p* < 0.01, ****p* < 0.001. vs. control media.

`
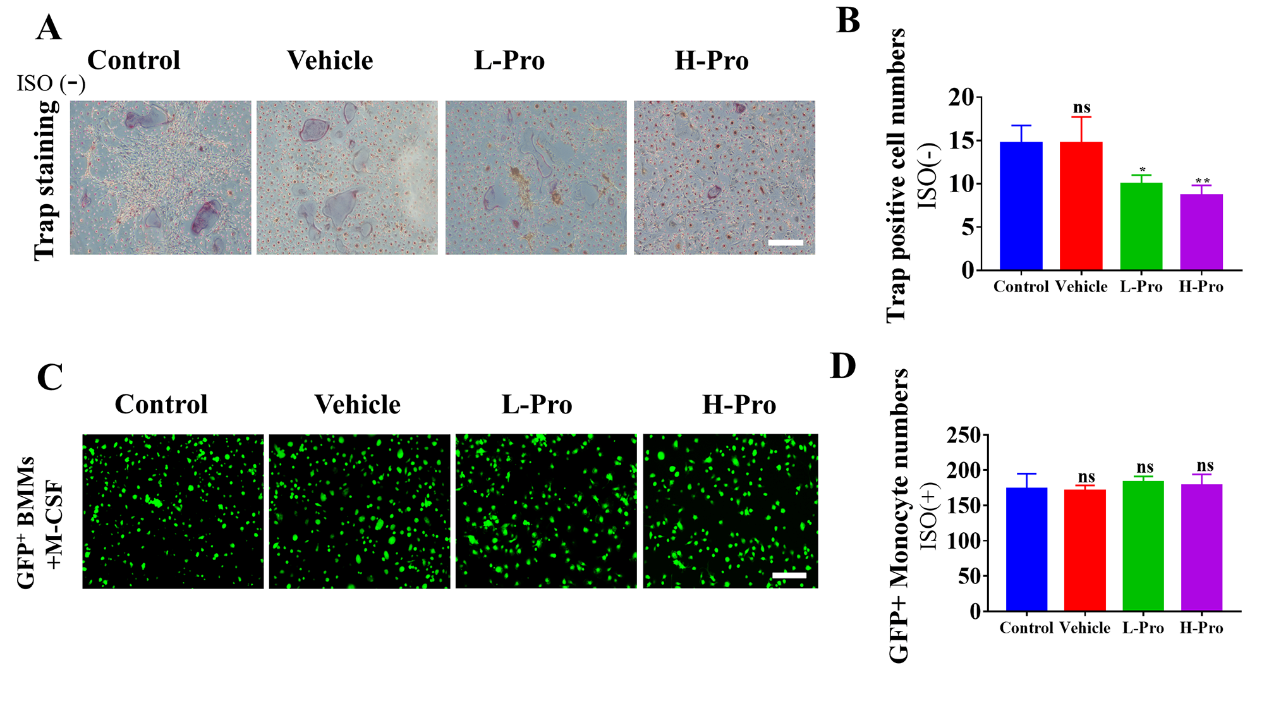


**Figure S5** (A) Trap staining of osteoclasts cultured in scaffold-conditioned media without isoprenaline. Scale bar: 200 μm. (B) Quantitative assessment of Trap^+^ cells, *n* = 3. (C) Representative images of GFP^+^ BMMs cultured in scaffold-conditioned media for 24 h in the presence of isoprenaline. Scale bar: 200 μm. (D) Quantification of GFP^+^ BMMs in (C), *n* = 3. Data are mean ± SD. ns: not significant; **p* < 0.05, ***p* < 0.01, ****p* < 0.001. vs. control.
